# Synergistic Effects of Dexamethasone and Dexmedetomidine in Extending the Effects of Pectoral I and Pectoral II Blocks for Postoperative Analgesia Following Total Mastectomy with Lymph Node Dissection

**DOI:** 10.3390/clinpract11020027

**Published:** 2021-03-30

**Authors:** Ahish Chitneni, Jamal Hasoon, Ivan Urits, Omar Viswanath, Alan D. Kaye, Jonathan Eskander

**Affiliations:** 1 Department of Anesthesiology, A.T. Still University School of Osteopathic Medicine in Arizona, Mesa, AZ 63501, USA; ahishchitneni@gmail.com; 2 Department of Anesthesiology, Critical Care, and Pain Medicine, Beth Israel Deaconess Medical Center, Harvard Medical School, Boston, MA 02115, USA; ivanurits@gmail.com; 3 Valley Anesthesiology and Pain Consultants, Envision Physician Services, Phoenix, AZ 85014, USA; viswanoy@gmail.com; 4 Department of Anesthesiology, University of Arizona College of Medicine Phoenix, Phoenix, AZ 85724, USA; 5 Department of Anesthesiology, Creighton University School of Medicine, Omaha, NE 68178, USA; 6 Department of Anesthesiology, Louisiana State University Health Shreveport, Shreveport, LA 71115, USA; akaye@lsuhsc.edu; 7 Anesthesiology and Pain Medicine, Portsmouth Anesthesia Associates, Portsmouth, VA 23707, USA; jeskande@tulane.edu

**Keywords:** regional anesthesia, acute pain, PECs block, ERAS

## Abstract

Regional anesthesia is an important aspect of the overall anesthetic plan for patients. It has the potential to offer superior benefits compared to opioid-based treatment and is an important component of Enhanced Recovery after Surgery (ERAS) protocols. The use of the pectoral type I (PECS I) and pectoral type II blocks (PECS II) has been shown to reduce postoperative pain and opioid consumption in patients undergoing complete mastectomy and breast surgery. We describe the use of dexamethasone and dexmedetomidine to prolong the analgesic effects of these regional blocks in a patient undergoing total mastectomy with lymph node dissection.

## 1. Introduction

Regional anesthesia is an important aspect of the overall anesthetic plan for patients. It has the potential to offer superior benefits compared to opioid-based treatment, reduce postoperative pain, and decrease the use of total opioid consumption in patients postoperatively [1]. Regional anesthesia for breast surgery has been traditionally administered via paravertebral nerve blocks or epidural analgesia. In recent years, the use of the pectoral type I (PECS I) and pectoral type II blocks (PECS II) has been shown to reduce postoperative pain and opioid consumption in patients undergoing complete mastectomy.

## 2. Case Description

Our patient was a 68-year-old Caucasian female with a past medical history of breast cancer, atrial fibrillation, osteoporosis, and major depressive disorder. The patient was diagnosed with cT2NOMO breast carcinoma with breast tumor characteristics demonstrating estrogen receptor (ER) and progesterone receptor (PR) positive and human epidermal growth factor receptor 2 (HER2) negative. After diagnosis, the patient completed neoadjuvant therapy with doxorubicin hydrochloride and cyclophosphamide, followed by treatment with paclitaxel before undergoing total mastectomy with lymph node dissection. Prior to the surgery, the patient underwent PECS I and PECS II blocks with 0.2% ropivacaine combined with 5 mg of preservative-free dexamethasone and 25 μg of dexmedetomidine. We administered 6 mL of the solution between the pectoralis muscles for the PECS I block, while another 10 mL was deposited between the serratus anterior and pectoralis minor muscles for the PECS II block. The surgery proceeded under general anesthesia maintained with volatile anesthetics with no additional opioids after 100 µg of fentanyl on induction.

The patient reported minimal postoperative pain after the surgery. She did not require any opioid pain medication for pain control while recovering in the PACU. Additionally, the patient reported that her pain was well controlled throughout her hospital stay. The patient reported pain scores ranging from 0 to 2/10 for five days after the procedure. She had minimal use of pain medications, which included acetaminophen, NSIADs, and low dose gabapentin on the first night after the surgery, but no opioid medication usage during the remainder of the postoperative course.

## 3. Discussion

The use of the PECS I block for providing postoperative analgesia in patients undergoing breast surgery was first described in 2011 by Blanco et al. as an alternative approach to both paravertebral and epidural blockade [2]. The technique describes the placement of local anesthetic between the pectoralis major and minor muscles. Since origination, Blanco et al. described a modification to the technique labeled the PECS II block or modified pecs block with the major difference being that the PECS I block targets the lateral pectoral nerves between the pectoralis major and minor muscles, whereas the PECS II block also targets the fascial plane between the serratus anterior and pectoralis minor muscles [3]. With the increased use of these blocks, there have been additional studies created to validate the efficacy of the pectoral blocks. Kim et al. conducted a prospective randomized controlled study in 2018, which compared patients receiving either the PECS II block to a control, and demonstrated that patients in the treatment group had a significantly lower opioid requirement and lower pain intensity [4]. Another retrospective study conducted in 2020 by Kim et al. demonstrated that the pectoral blocks reduced perioperative opioid consumption after mastectomy surgery compared to patients receiving general anesthesia alone [5]. Reports have also demonstrated the utility of utilizing either dexamethasone or dexmedetomidine during regional anesthesia to provide prolonged postoperative analgesia in a variety of surgical cases [6,7]. Furthermore, there is recent evidence that the combination of these adjuvants together during regional anesthesia may provide even longer postoperative analgesia and should be considered for patients undergoing particularly painful surgical procedures [8,9].

## 4. Conclusions

The utilization of regional anesthesia has clear benefits in reducing postoperative pain and opioid consumption in patients undergoing surgeries such as mastectomy with lymph node dissection. Given the postoperative pain relief seen with PECS I and PECS II blocks, the technique may be an important aspect of the pain regimen for patients undergoing similar surgical procedures. Additionally, this report demonstrates the utility of combining dexamethasone and dexmedetomidine to the PECS I and PECS II nerve blocks and adds to the growing literature suggesting increased benefit with adjuvants during regional anesthesia. To date, there are mostly case reports and case series suggesting the benefits of utilizing both dexamethasone and dexmedetomidine for prolonged analgesia in fascial plane blocks. To our knowledge, this is among the first reports to describe combined dexamethasone and dexmedetomidine in PECS I and PECS II blocks. Further studies should be conducted to explore the safety and efficacy of the combination of dexmedetomidine and dexamethasone as adjuvants to local anesthetic in regional anesthesia.

## Data Availability

No new data were created or analyzed in this study. Data sharing is not applicable to this article.
